# Involvement of AF1q/MLLT11 in the progression of ovarian cancer

**DOI:** 10.18632/oncotarget.15574

**Published:** 2017-02-21

**Authors:** Paola Tiberio, Ludmila Lozneanu, Valentina Angeloni, Elena Cavadini, Patrizia Pinciroli, Maurizio Callari, Maria Luisa Carcangiu, Domenica Lorusso, Francesco Raspagliesi, Valentina Pala, Maria Grazia Daidone, Valentina Appierto

**Affiliations:** ^1^ Department of Experimental Oncology and Molecular Medicine, Biomarkers Unit, Fondazione IRCCS Istituto Nazionale dei Tumori, Milan, Italy; ^2^ Department of Morphofunctional Sciences–Histology, Patology, “Grigore T. Popa” University of Medicine and Pharmacy, Iassy, Romania; ^3^ Department of Experimental Oncology and Molecular Medicine, Functional Genomics Facility, Fondazione IRCCS Istituto Nazionale dei Tumori, Milan, Italy; ^4^ Present address: Cancer Research UK Cambridge Institute, University of Cambridge, Cambridge, United Kingdom; ^5^ Department of Pathology, Fondazione IRCCS Istituto Nazionale dei Tumori, Milan, Italy; ^6^ Department of Surgery, Gynecologic Oncology Unit, Fondazione IRCCS Istituto Nazionale dei Tumori, Milan, Italy

**Keywords:** AF1q/MLLT11, ovarian cancer, borderline ovarian tumor/BOT, low malignant potential ovarian tumor, EMT

## Abstract

The functional role of AF1q/MLLT11, an oncogenic factor involved in a translocation t(1;11)(q21;q23) responsible for acute myeloid leukaemia, has been investigated in hematological and solid malignancies and its expression was found to be linked to tumor progression and poor clinical outcome. In addition to its oncogenic function, AF1q has been shown to play a role in the onset of basal and drug-induced apoptosis in cancer cells of different histotypes, including ovarian cancer. Through *in vitro*, *ex vivo*, and *in silico* approaches, we demonstrated here that AF1q is also endowed with protumorigenic potential in ovarian cancer. In ovarian cancer cell lines, stable AF1q overexpression caused activation of epithelial-to-mesenchymal transition and increased motility/migratory/invasive abilities accompanied by gene expression changes mainly related to Wnt signaling and to signaling pathways involving in ERK/p38 activation. The potential role of AF1q in ovarian cancer progression was confirmed by immunohistochemical and *in silico* analyses performed in ovarian tumor specimens which revealed that the protein was absent in normal ovarian epithelium and became detectable when atypical proliferation was present. Moreover, AF1q was significantly lower in borderline ovarian tumors (i.e., tumors of low malignant potential without stromal invasion) than in invasive tumors, thus corroborating the association between high AF1q expression and increased migratory/invasive cell behavior and confirming its potential role in ovarian cancer progression. Our findings demonstrated, for the first time, that AF1q is endowed with protumorigenic activity in ovarian cancer, thus highlighting a dual behavior (i.e., protumorigenic and proapoptotic functions) of the protein in the malignancy.

## INTRODUCTION

The ALL1-fused from chromosome 1q (AF1q or MLLT11) gene, located on chromosome 1q21, encodes a small protein of 90 amino acids (9 kDa) with no well-defined functional domains, no significant similarity to other proteins and no clarified biological functions [1]. AF1q was originally identified as an oncogenic factor implicated in a translocation t(1;11)(q21;q23) involved in the development of acute myeloid leukaemia (AML). Moreover, the AF1q locus has been involved in complex events of translocation and duplication in other hematological malignancies [2, 3]. In the absence of specific cytogenetic alterations, elevated AF1q mRNA expression has been reported in lymphoid and myeloid malignancies [2, 4, 5] and has been associated with a poor prognosis in pediatric AML and also in adult patients with myelodysplastic syndrome [6, 7]. An oncogenic function of AF1q has been reported also in certain solid tumors, such as thyroid oncocytic and testicular germ cell tumors [8, 9] and, in breast cancer, it has been shown to promote distant metastasis [10–12], although the molecular mechanisms underlying this function have not yet been fully elucidated. Important advances in understanding the biological functions of AF1q associated with breast cancer metastasis have recently been obtained by Park and colleagues [12, 13]. The authors demonstrated that AF1q acts as cofactor for both Wnt and STAT signaling pathways, via direct interaction with T-cell-factor-7 and activation of *Src*-platelet-derived growth factor subunit B kinase cascade, respectively. In both cases, the binding of AF1q to transcription factors results in transcriptional activation of genes required for tumorigenesis and metastasis.

In addition to AF1q oncogenic functions, there is also evidence of a role as an apoptosis mediator in hepatocellular, ovarian and squamous carcinoma and in promyelocytic leukemia cells [14–16]. Thus, similarly to that reported for certain oncogenes (i.e., Myc and Ras) [17], AF1q has been shown to be endowed with a dual function in malignancy, being a protein apparently involved in both promotion and inhibition of cancer progression.

To gain further insight into the role of AF1q in tumorigenesis of solid malignancies, we investigated the protumorigenic potential of the protein in ovarian cancer. The malignancy is the fifth most common female cancer, with an incidence of about 3% of all cancers in women, and epithelial ovarian cancer is the most common type, accounting for 90% of all ovarian neoplasms [18]. Despite the considerable advances made in ovarian cancer management and the intensive efforts to elucidate predisposing factors, the malignancy remains one with the lowest survival rates, with a mortality rate higher than any other cancer of the female reproductive system. Rapid disease progression and metastasis development represent critical factors for a poor clinical outcome and cancer mortality. In fact, due to the absence of symptoms in the early stages of the disease, most patients are diagnosed when tumors have local or distant spread [18].

We herein report, for the first time, that AF1q plays a role in promoting epithelial-to-mesenchymal transition (EMT) and the acquisition of an invasive and aggressive behavior of cultured ovarian cancer cells and that elevated expression of the protein is associated with clinical aggressiveness and progression in ovarian cancer specimens.

## RESULTS

### Sustained AF1q overexpression induces a spindle-shape phenotype and cytoskeleton rearrangement in A2780 ovarian cancer cells

**Figure 1:**
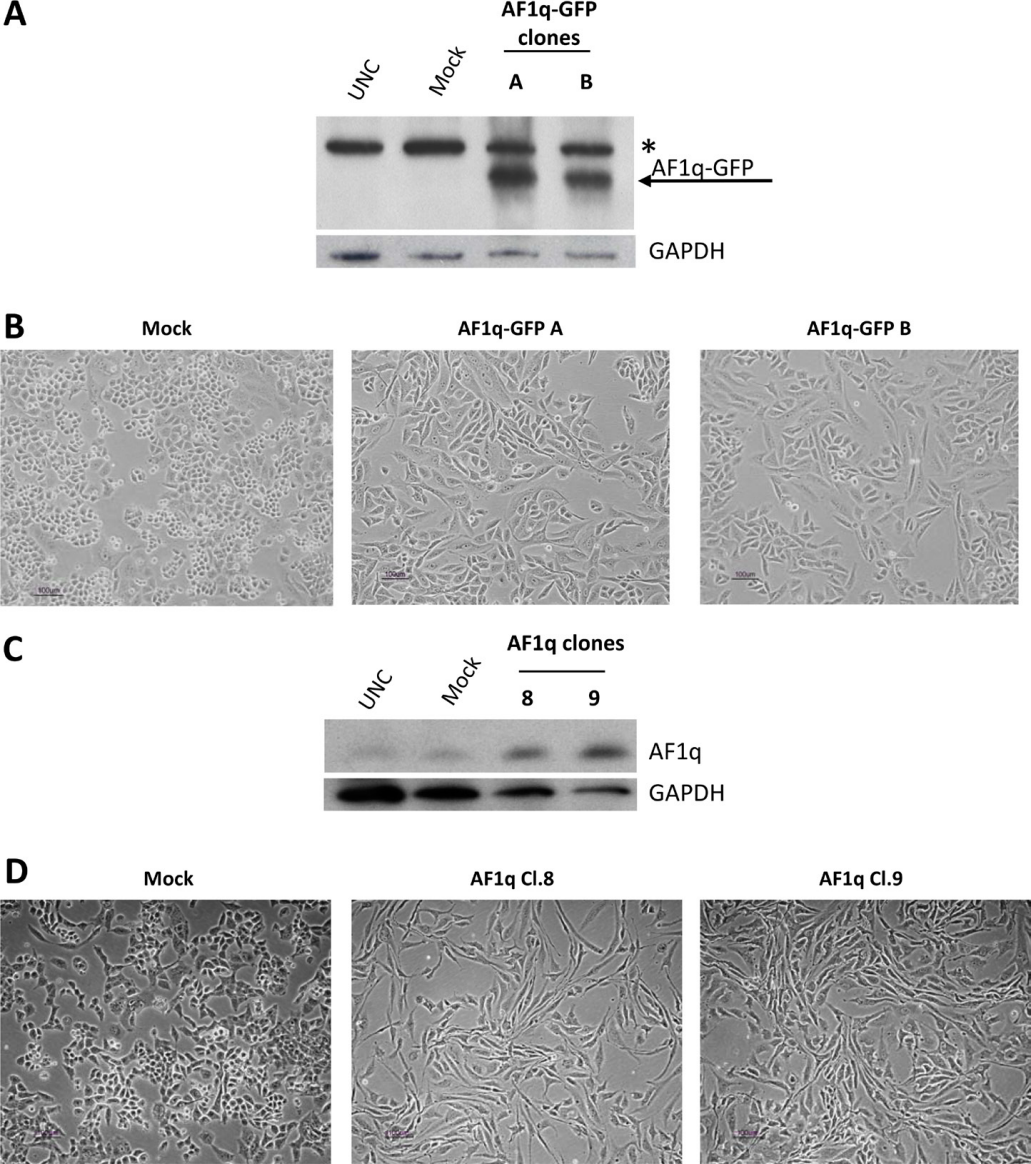
AF1q stable overexpression induced a spindle shape cell phenotype in A2780 ovarian cancer cells (**A**) Western blot showing the expression of AF1q-GFP fusion protein in A2780 cells stably transfected with a GFP-tagged AF1q vector (AF1q-GFP clones A and B) as compared to cells transfected with GFP vector (mock) and untransfected cells (UNC). The blot was incubated with GAPDH antibody, as loading control. The star indicates a non-specific band. (**B**) Morphological appearances of mock and AF1q-GFP A2780 clones. (**C**) Western blot showing AF1q protein expression in A2780 cells stably transfected with a plasmid containing the full-length AF1q coding region (clones 8 and 9) as compared to cells transfected with an empty vector (mock) and untrasfected cells (UNC). As a control for loading, the blot was incubated with GAPDH antibody. (**D**) Morphological appearances of mock and AF1q overexpressing A2780 clones.

In addition to the oncogenic function of AF1q described in hematological as well as in solid malignancies [6–11, 19], some studies have shown that elevated expression levels of the protein were associated to increased apoptosis in cancer cells [14–16]. In the present study, we investigated whether the protein is also endowed with an oncogenic potential in ovarian cancer. The A2780 ovarian cancer cell line was stably transfected with a Green Fluorescent Protein (GFP)-tagged AF1q expression plasmid previously shown to increase apoptosis when transiently transfected in the cell line [16]. After geneticin selection, resistant clones were isolated and analyzed by western blot to confirm the presence of the AF1q-GFP protein. Figure 1A reports the results of two representative clones (A and B) which presented higher expression of the recombinant protein than that expressed by the mock clone (empty vector). Overexpression of the AF1q-GFP protein caused a dramatic change in cell morphology from a round and cuboidal cell shape to a long, spindle, fibroblast-like cell appearance (Figure 1B). To be sure that the observed changes of cell phenotype were attributable to the increased expression of AF1q and not to an unwanted alteration of protein functions due to the fusion of AF1q (9 kDa) to GFP (36 kDa), new stable transfectants were established using an expression vector (pcDNA 3.1) containing the AF1q open reading frame without GFP. Figure 1C reports the results of western blot analysis of two representative clones (Cl.8 and 9) showing AF1q overexpression relative to mock clone or A2780 parental cells. Similarly to that observed in clones A and B, AF1q stable Cl.8 and Cl.9 displayed a phenotype more elongated and a spindle-like shape than that of the mock clone (Figure 1D). Since AF1q-GFP and AF1q-overexpressing clones showed very similar morphological features, we can exclude that the effect was caused by the fusion protein AF1q-GFP. The following analyses were performed only in Cl.8 and Cl.9 clones.

**Figure 2:**
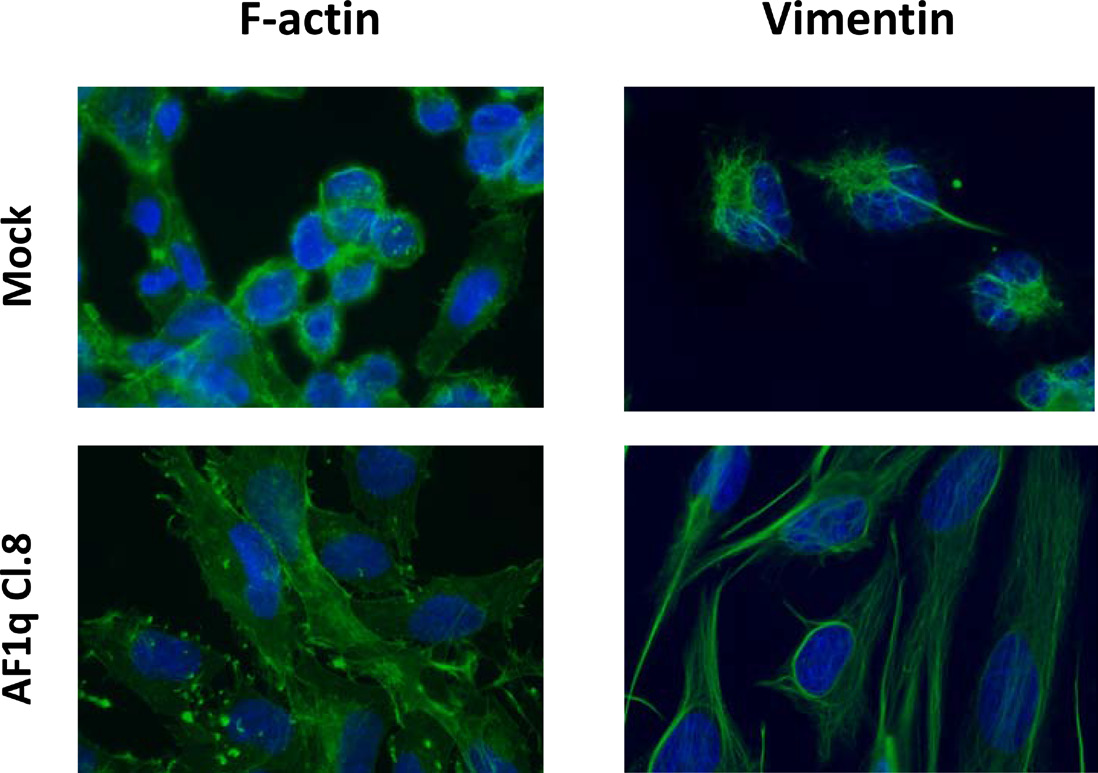
AF1q overexpression induced cytoskeleton remodeling in A2780 ovarian cancer cells Microscopic immunofluorescent visualization of F-actin and vimentin in A2780 cells stably overexpressing AF1q (Cl.8) as compared to mock cells. Photos were taken at 50× magnification.

To further characterize the morphological alterations induced by AF1q overexpression, the arrangement of filamentous actin (F-actin) and vimentin fibers contributing to the cytoskeletal structure were analyzed. Immunofluorescence analysis revealed that AF1q-overexpressing clones displayed a modified organization of both filaments, showing a remarkable increase in membrane actin ruffles and a reorganization of vimentin, from short and focally localized fibers to a long, dense, and parallel network (Figure 2, data shown only for Cl.8). Such results indicated that stable AF1q overexpression in A2780 cells induced a morphological alteration, leading to a spindle-like phenotype, and cytoskeleton reorganization that may be suggestive of an increased migratory capacity.

### AF1q overexpression promotes cell motility, migration and invasion of A2780 ovarian cancer cells

**Figure 3:**
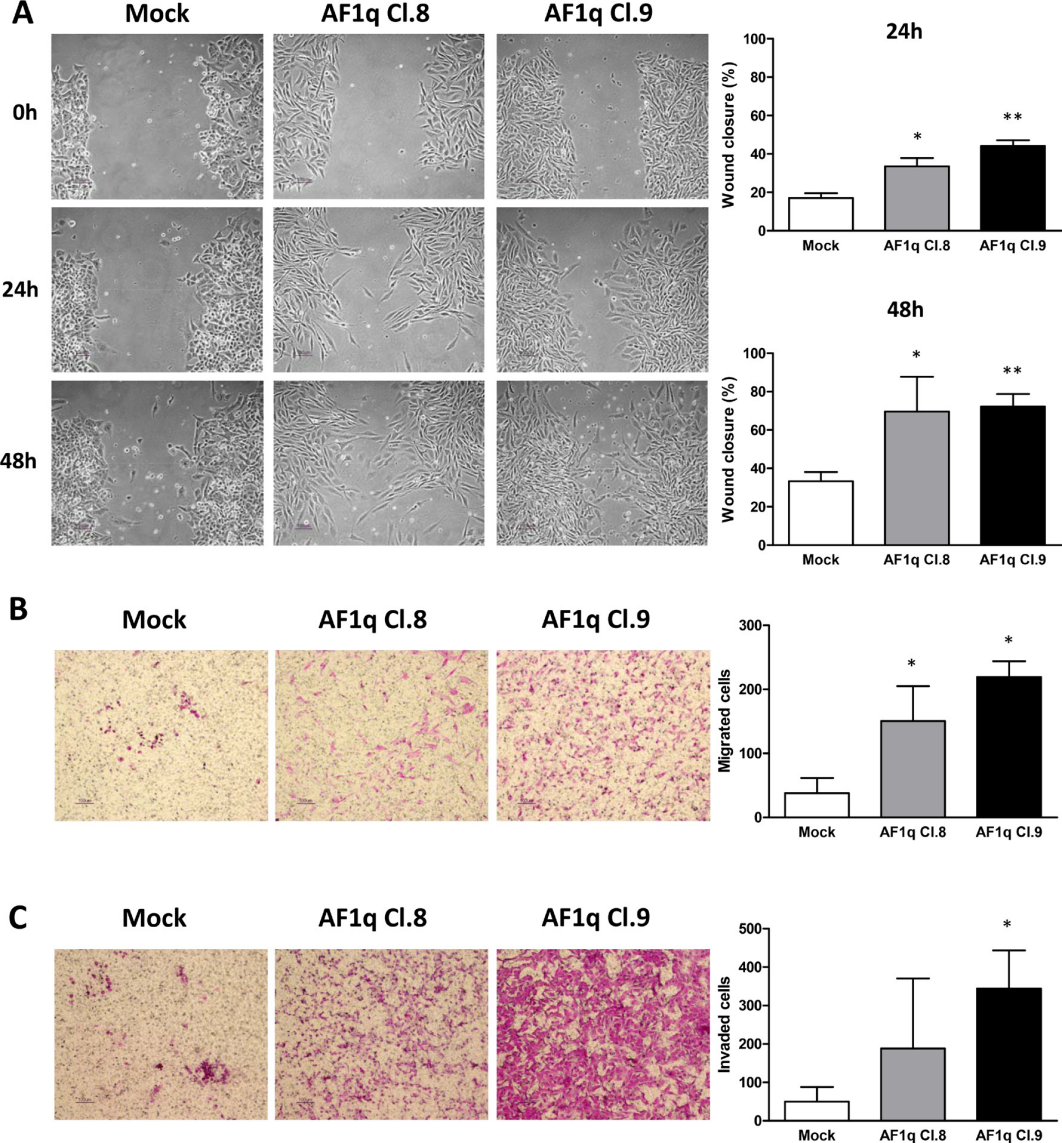
AF1q stable overexpression promotes cell motility and migration in A2780 ovarian cancer cells Whound-healing (**A**), migration (**B**) and invasion (**C**) assays evaluating the change of cell mobility in A2780 cells stably overexpressing AF1q (AF1q Cl.8 and 9) compared to mock cells. (A) Representative images of wound healing assays evaluated at 24 and 48 h after scratch. Graphs represent the quantification of “gap closure” (Cl.8: *p* = 0.013 and 0.049 at 24 and 48 h, respectively; Cl.9: *p* = 0.006 and 0.002 at 24 and 48 h, respectively). (B) and (C) Representative images and graphs relative to transwell migration (*p* = 0.027 and 0.015 for Cl.8 and Cl.9, respectively) and invasion (*p* = 0.024 for Cl.9) assays, evaluated at 48 h, graphs represent the relative migration ability calculated from at least 4 fields under a light microscope. The data are represented as mean ± S.D. from three independent experiments.

The effect of AF1q stable overexpression in cell migration was assessed by performing wound-healing and transwell assays. The wound-healing assays showed that, after 24 as well as 48 h from the monolayer scratch, Cl.8 and Cl.9 cells were both able to close the wound more efficiently than were the mock cells (Figure 3A) (Cl.8: *p* = 0.013 and 0.049 at 24 and 48 h, respectively; Cl.9: *p* = 0.006 and 0.002 at 24 and 48 h, respectively). Similar results were obtained when migration ability was tested through transwell assays, which showed that migration and invasion of AF1q-overexpressing clones were increased compared to those of control cells (Figure 3B). Specifically, migration of Cl.8 and Cl.9 was ~4 and ~6 fold higher than that of the mock clone (*p* = 0.027 and 0.015, respectively), whereas invasion was enhanced by ~4 and ~7 fold, respectively, that of the mock clone (Cl.9: *p* = 0.024, whereas the increase was not statistically significant for Cl.8: *p* = 0.24) (Figure 3C). Such results indicated that stable overexpression of AF1q increased the motility and migratory/invasive abilities of A2780 cells.

**Figure 4:**
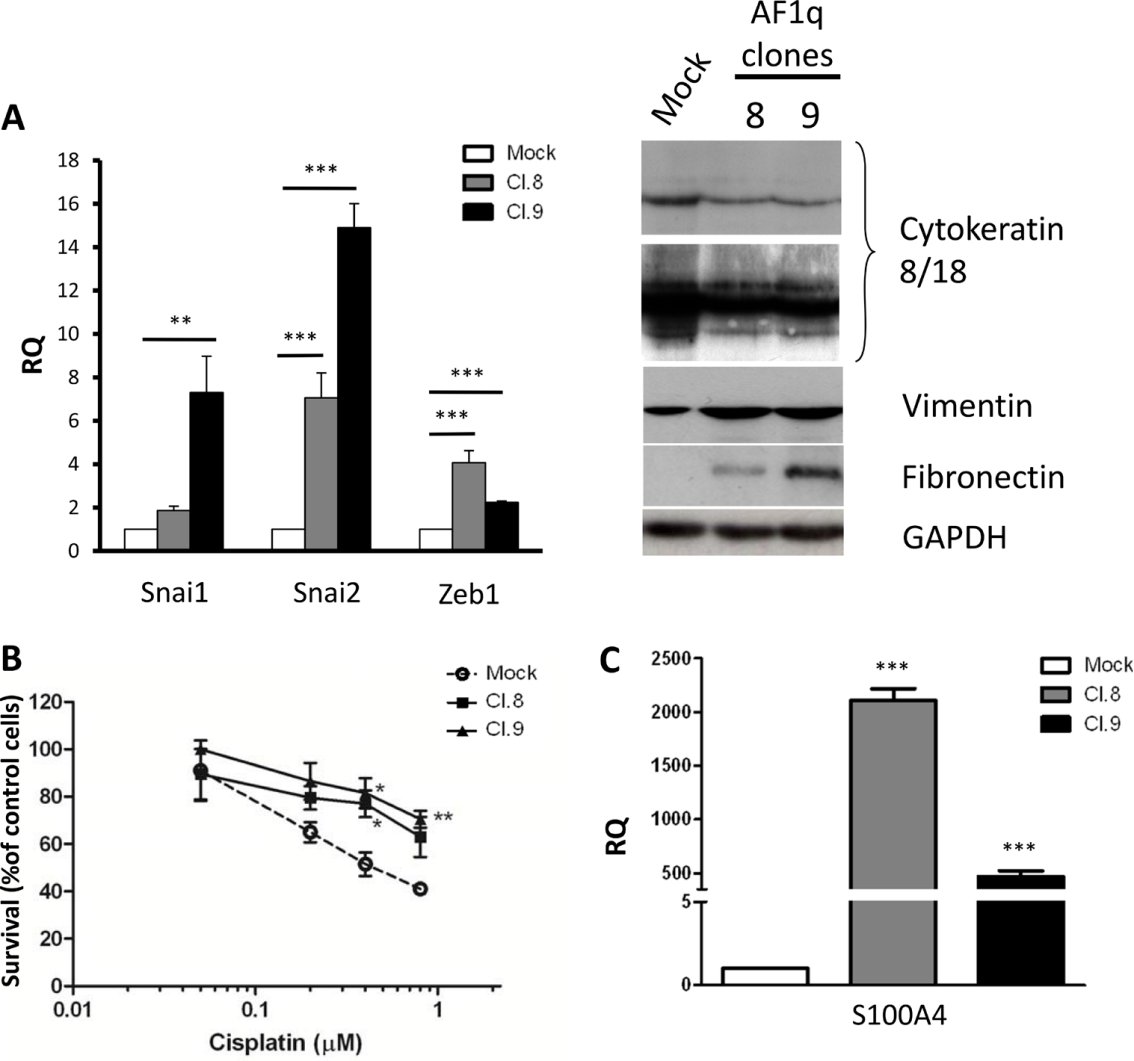
AF1q overexpression induce acquisition of mesenchymal traits in A2780 ovarian cancer cells (**A**) Real-Time PCR analyses of Snai1, Snai2 and Zeb1 mRNA expression levels normalized to Gapdh mRNA level (used as an internal control) (left panel) and Western blot analysis for the expression of cytokeratins 8 and 18, vimentin, and fibronectin (right panel) in A2780 cells stably overexpressing AF1q (Cl.8 and Cl.9) compared to mock cells. As a control for loading, the blot was incubated with GAPDH antibody. (**B**) Growth inhibition assay in the presence of increasing concentrations of cisplatin (from 0.05 μM to 0.8 μM). (**C**) Real-Time PCR analyses of S100A4 mRNA levels normalized to Gapdh (used as an internal control). Asterisk indicate *p*-value: ** from 0.001 to 0.01 and ***< 0.001

The spindle-shaped morphology and the increased migratory/invasive capacity acquired by A2780 cells stably transfected with AF1q may be indicative of EMT. Consistent with this hypothesis, Real-Time PCR analyses revealed that Cl.8 and Cl.9 cells, compared to mock cells, both displayed an increased expression of the EMT-related transcription factors Snai1, Snai2 and Zeb1 (Figure 4A). Moreover, Western blot analysis showed that AF1q-overexpressing clones were concomitantly characterized by a reduced expression of the epithelial markers, cytokeratins 8 and 18, and increased expression of the mesenchymal markers vimentin and fibronectin (Figure 4A). In this particular cell line, we could not evaluate EMT activation based on the down-regulation of E-cadherin, the classical hallmark of the process, because A2780 cells did not express the protein (data not shown).

Acquisition of mesenchymal traits by tumor cells has been associated not only to invasive/metastatic ability but also to drug resistance. Since in ovarian cancer a link between EMT and resistance to platinum-based chemotherapy has been reported [20], we investigated whether AF1q overexpression caused change in cell sensitivity to the drug. As shown in Figure 4B, Cl.8 and Cl.9 cells, compared to mock cells, both displayed a decreased sensitivity to cisplatin growth inhibitory activity: a 50% growth inhibition was achieved with 0.46 μM cisplatin in mock cells, whereas the IC_50_ values (concentrations required for 50% growth inhibition) of this drug were 2.2 and 2 μM for Cl.8 and Cl.9 cells, respectively.

Taken together, the *in vitro* experiments conducted on A2780 cells might suggest an involvement of AF1q in ovarian tumor progression and resistance to chemotherapy.

### Gene expression analysis confirmed a role of AF1q in EMT and indicated Wnt signaling and MAPK cascade as AF1q mediators

To explore the molecular pathways involved in AF1q activity, we analyzed the changes in gene expression induced by AF1q overexpression in A2780 cells. Gene expression profiles of Cl.9 and mock cells were compared by microarray analysis and 1804 genes (i.e., 916 up-regulated and 888 down-regulated in Cl.9 cells; adj *p*-value < 0.001 and at least a two-fold difference between the two conditions) were found differentially expressed (Supplementary Figure 1). Interesting observations emerged by looking at single gene level (the top 25 up and down-regulated genes are listed in Supplementary Table 1). For example, among the top up-regulated genes, we found genes playing a role in invasiveness and metastasis (i.e., MMP3 [21] and MMP10 [22, 23], SOX18 [24], MARCKS [25], CD36 [26] and BST2 [27]). A marked up-regulation of AKR1 enzymes (AKR1C2, AKR1C3 and AKR1C4) which are involved in steroid hormones metabolism [28], including estrogen biosynthesis, was also observed. Intriguingly, the second most up-regulated gene by AF1q transfection was S100A4. This gene is a direct target of β-catenin/T-cell factor signaling, playing an important role in the acquisition of aggressive characteristics in ovarian carcinomas [29]. S100A4 has been shown to act as a master mediator of EMT and to modulate the sensitivity to anticancer drugs, including cisplatin [30]. Real-Time PCR analysis of S100A4 expression, extended also to Cl.8, confirmed its marked increase in AF1q transfected cells, compared to mock cells (Figure 4C). On the other hand, genes suppressing migration (DIRAS3 [31]) and metastasis (RBM47 [32] and CRB3 [33]) were found among the most down-regulated genes.

**Figure 5:**
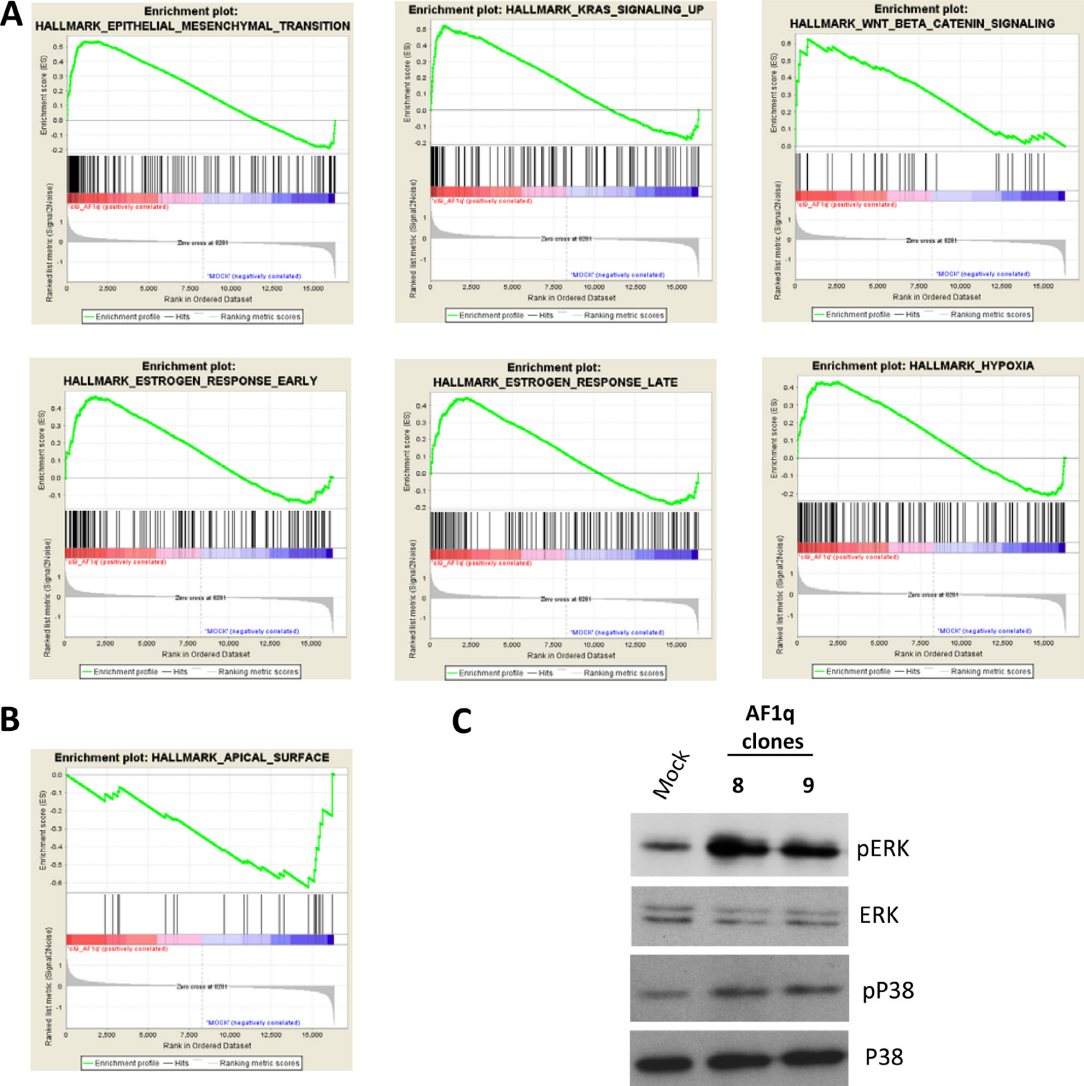
Molecular pathways involved in AF1q activity Selected gene sets positively (**A**) and negatively (**B**) enriched in AF1q transfected cells obtained by GSEA hallmark analysis. (**C**) Western blot analysis of the phosphorylated (active form) ERK and p38 in A2780 cells stably overexpressing AF1q (Cl.8 and Cl.9) compared to mock cells. Total ERK and p38 were used as loading control.

AF1q-induced changes in gene expression were then analyzed by Gene Set Enrichment Analysis (GSEA), using the Hallmark Gene Set Collection [34]. Positively (*n* = 9) and negatively (*n* = 1) enriched gene sets (FDR < 0.25) are listed in Supplementary Table 2, and selected gene set enrichment plots are shown in Figure 5A and 5B. Not surprisingly, the EMT gene set was positively enriched in AF1q transfected cells (Figure 5A). At the same time, a set of genes typically expressed in the apical surface of epithelial cells was negatively enriched (Figure 5B). These findings are fully consistent with the acquisition of mesenchymal traits in culture. A positive enrichment was also found for genes in the Wnt/β-catenin pathway. This observation was in line with the above mentioned S100A4 induction and with the recent identification of AF1q as a TCF7/LEF1 co-factor acting downstream the Wnt signaling pathway in breast cancer metastatization [12].

Interestingly, also gene sets related to Ras signaling, hypoxia and estrogen response were positively enriched in AF1q transfected cells (Figure 5A). Besides their common involvement in EMT, invasion and metastasis processes, several studies reported that these pathways are linked to the activation of the mitogen-activated protein kinases (MAPKs) ERK and p38 [35–37]. Therefore, we investigated whether AF1q overexpression led to ERK and p38 activation in A2780 cells, determining their phosphorylation status. Western blot using phospho-specific antibodies detecting the phosphorylated (active) forms of the kinases showed increased kinase phosphorylation in Cl.8 and Cl.9 compared to mock cells (Figure 5C).

Taken together, these results confirmed at gene expression level a role of AF1q in EMT and in tumor progression of ovarian cancer cells and indicated Wnt signaling and MAPK cascade as potential mediators.

### AF1q is involved in EMT transition in OVCAR-5 and TOV-21G ovarian cancer cells

**Figure 6:**
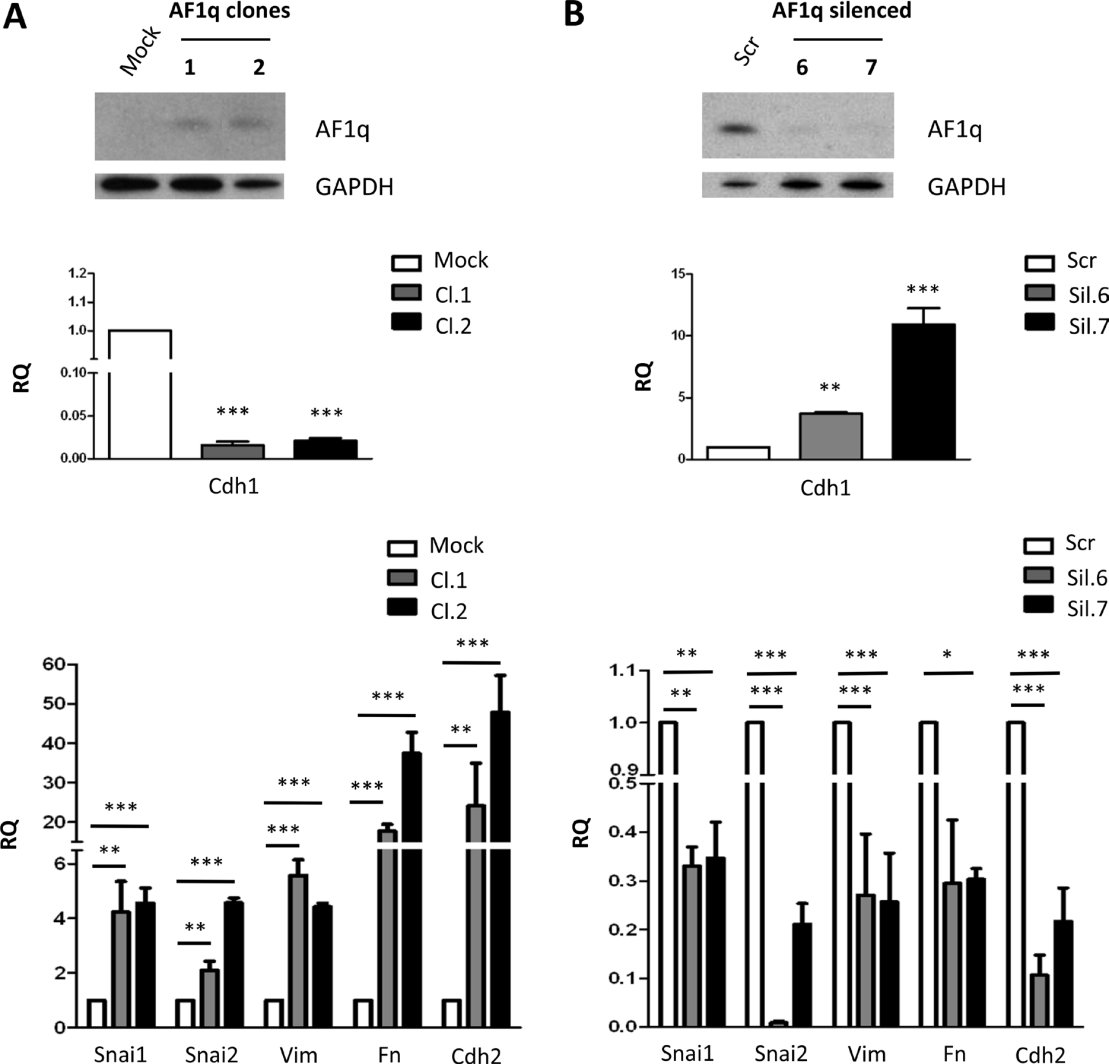
AF1q is involved in EMT transition in OVCAR-5 and TOV-21G ovarian cancer cells (**A**) Western blot showing AF1q protein overexpression in OVCAR-5 cells stably transfected with a plasmid containing AF1q full-length coding sequence (AF1q clones 1 and 2) compared to cells transfected with empty vector (Mock). The blot was incubated with GAPDH antibody as loading control. Real-Time PCR analysis of Cdh1 (middle panel) and Snai1, Snai2, vimentin (Vim), fibronectin (Fn) and Cdh2 (lower panel) normalized to Gapdh mRNA level, as internal control. (**B**) Western blot showing AF1q protein silencing in TOV-21G cells stably silenced with a plasmid containing sh-RNA for AF1q (AF1q silenced 6 and 7) compared to cells transfected with a vector containing a scramble sh-RNA (Scr). The blot was incubated with GAPDH antibody as loading control. Real-Time PCR analysis of Cdh1 (middle panel) and Snai1, Snai2, vimentin (Vim), fibronectin (Fn) and Cdh2 (lower panel) expression normalized to Gapdh mRNA level, as internal control. Asterisk indicate *p*-value: * from 0.01 to 0.05, ** 0.001 to 0.01 and ***< 0.001.

To assess whether AF1q involvement in the EMT process was restricted to A2780 cells or represented a distinctive function of the gene in ovarian cancer cells, we extended our analysis to two other ovarian cancer cell lines, OVCAR-5 and TOV-21G. OVCAR-5 cells, which have undetectable AF1q expression, were stably transfected with AF1q. After geneticin selection, two resistant clones (Cl.1 and Cl.2) were isolated and analyzed by Western blot, which confirmed the presence of the protein (Figure 6A). Similarly to that observed in A2780 cells, AF1q overexpression conferred an elongated morphology to OVCAR-5 cells (Supplementary Figure 2) and acquisition of mesenchymal markers. In fact, enforced AF1q expression resulted in down-regulation of E-cadherin, in increased N-cadherin, vimentin and fibronectin as well as Snai1 and Snai2 mRNA levels (Figure 6A). According to the EMT-promoting function of AF1q observed in A2780 and OVCAR-5 cells, suppression of endogenous AF1q expression in TOV-21G cells resulted in a decrease of mesenchymal markers (Figure 6B), although no evident changes in morphology were observed (data not shown). These findings indicated that the role of AF1q in EMT was not restricted to A2780 cells but could be reliably considered as a more general mechanism.

Finally, we investigated whether the acquisition of epithelial traits caused *per se* a decrease in AF1q expression level. To this aim TOV-21G and A2780 cells were transiently co-transfected with miR-200c and miR-141, two members of the miR-200 family, a master regulator of MET targeting ZEB1 3′UTR [38], a repressor of the E-cadherin transcription [39]. After ascertain that miRNA transfection resulted in increased levels of miR-200c and miR-141 (Supplementary Figure 3A), we verified that, as expected, the expression level of ZEB1 and E-cadherin were decreased and increased, respectively (Supplementary Figure 3B). In both cell lines the acquisition of epithelial traits did not affect AF1q expression (Supplementary Figure 3B), thus suggesting that AF1q acts as an upstream regulator of EMT rather than an effector molecule.

### AF1q expression is related to ovarian tumor malignancy

**Figure 7:**
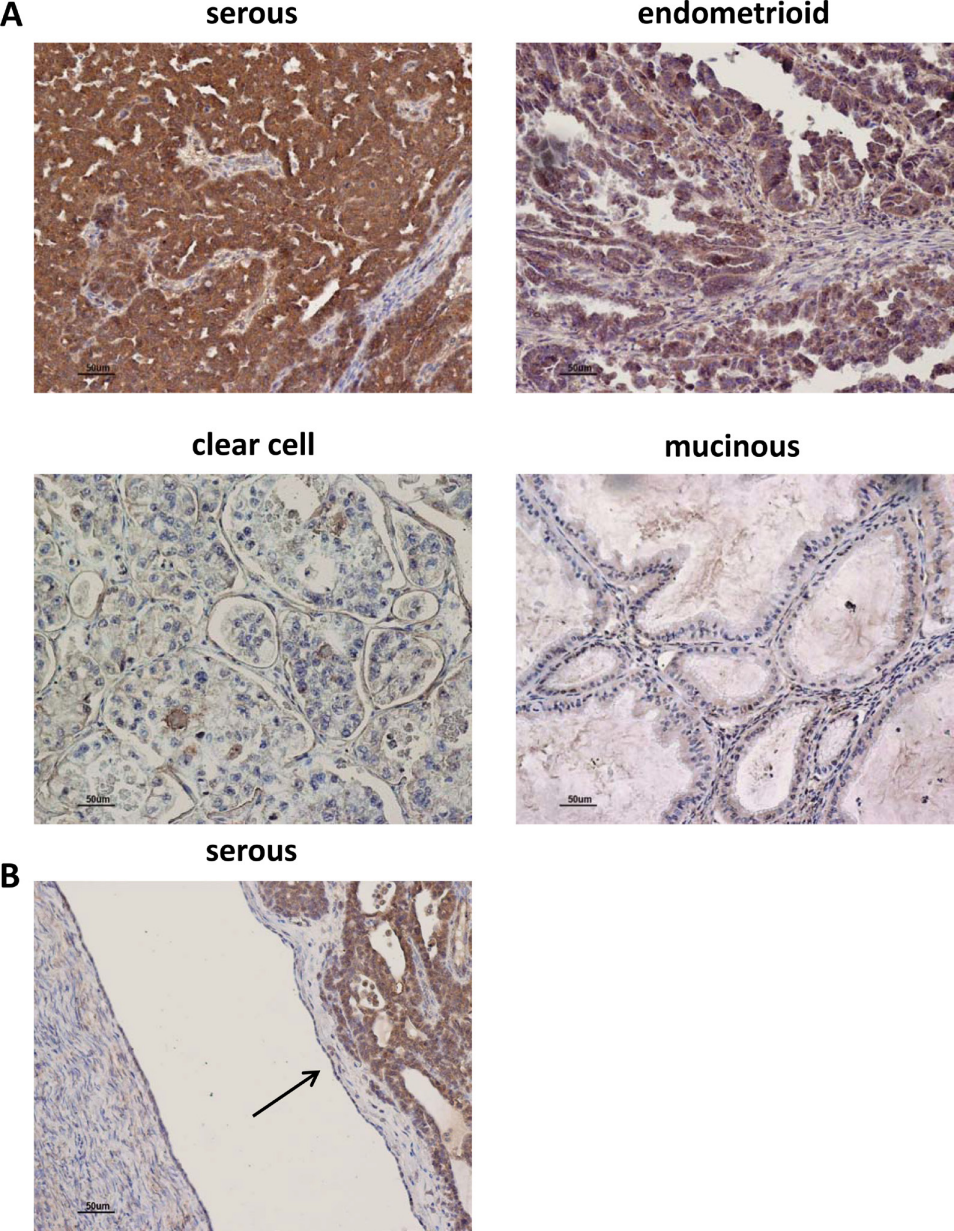
AF1q immunostaining in human ovarian tumor tissues (**A**) Representative images of AF1q staining in human ovarian tumors of different hystotypes. High IHC staining of AF1q in serous and endometrioid tumors. Low and negative IHC staining of AF1q in clear cell, and mucinous tumors, respectively. (**B**) Representative image of negative IHC staining of AF1q in OSE cells indicated by the arrow.

AF1q expression was analyzed by IHC in 47 primary invasive ovarian tumors (Table 1). This analysis showed an AF1q positive immunostaining in tumor epithelial cells of serous, endometrioid, clear cells, and undifferentiated carcinomas, but not of the mucinous type. In particular, serous and endometroid carcinomas showed AF1q expression higher than the other histotypes (Figure 7A and Table 1). In AF1q-positive specimens, the immunostaining was present in tumor cells, predominantly localized in the cytoplasm and characterized by a granular pattern. By contrast, AF1q expression was undetectable in stroma and in normal ovarian surface epithelium (OSE) (Figure 7B). However, in specific areas of OSE, showing stratified epithelial proliferation characterized by mitotic activity and nuclear atypia, AF1q staining was detectable (data not shown). No statistically significant differences were found between AF1q expression levels and tumor stage or grade. In this analysis, stage III (i.e., cancer has spread to the peritoneum outside the pelvis and/or metastatized to the retroperitoneal lymph nodes) and stage IV (i.e., cancer has spread to distant sites) were grouped together due to the paucity of stage IV cases (*n* = 3). On the contrary, looking at the pathological classification in type I and type II ovarian cancers, a statistically significant association between type II ovarian cancers and high AF1q expression levels was observed (*p* = 0.0447, Table 1). Type I tumors are generally slow-growing, confined to the ovary and comprise: low-grade serous, low-grade endometrioid, clear-cell, mucinous, and transitional tumors. Type II tumors are typically highly aggressive, rapidly proliferating and associated with a poor prognosis; they include: high-grade serous and endometrioid carcinomas and undifferentiated carcinomas.

**Table 1:** Invasive ovarian cancer patients’ characteristics

Variables	Patients *n* = 47	AF1q		*p* value
		Low (%)	High (%)	
Age				
≤ 55	20	9 (45)	11 (55)	
> 55	27	5 (18.5)	22 (81.5)	0.0617
Tumor histotype				
Serous*	31	6 (19.4)	25 (80.6)	0.0447*
Endometrioid^*^	9	2 (22.2)	7 (77.8)	0.0016**
Clear Cell	2	2 (100)	0 (0)	
Mucinous	4	4 (100)	0 (0)	
Undifferentiated	1	0 (0)	1 (100)	
FIGO Stage				
I/II	22	8 (36.4)	14 (63.6)	
III/IV	25	6 (24)	19 (76)	0.5238
Tumor Grade				
G1/G2	15	6 (40)	9 (60)	
G3/G4	32	8 (25)	24 (75)	0.3239
Type Classification				
Type I	16	8 (50)	8 (50)	
Type II	31	6 (19.4)	25 (80.6)	**0.0447**

Data in bold indicate *p* < 0.05.

^*^Serous versus non-serous.

^**s^Serous and endometrioid versus all others.

**Figure 8:**
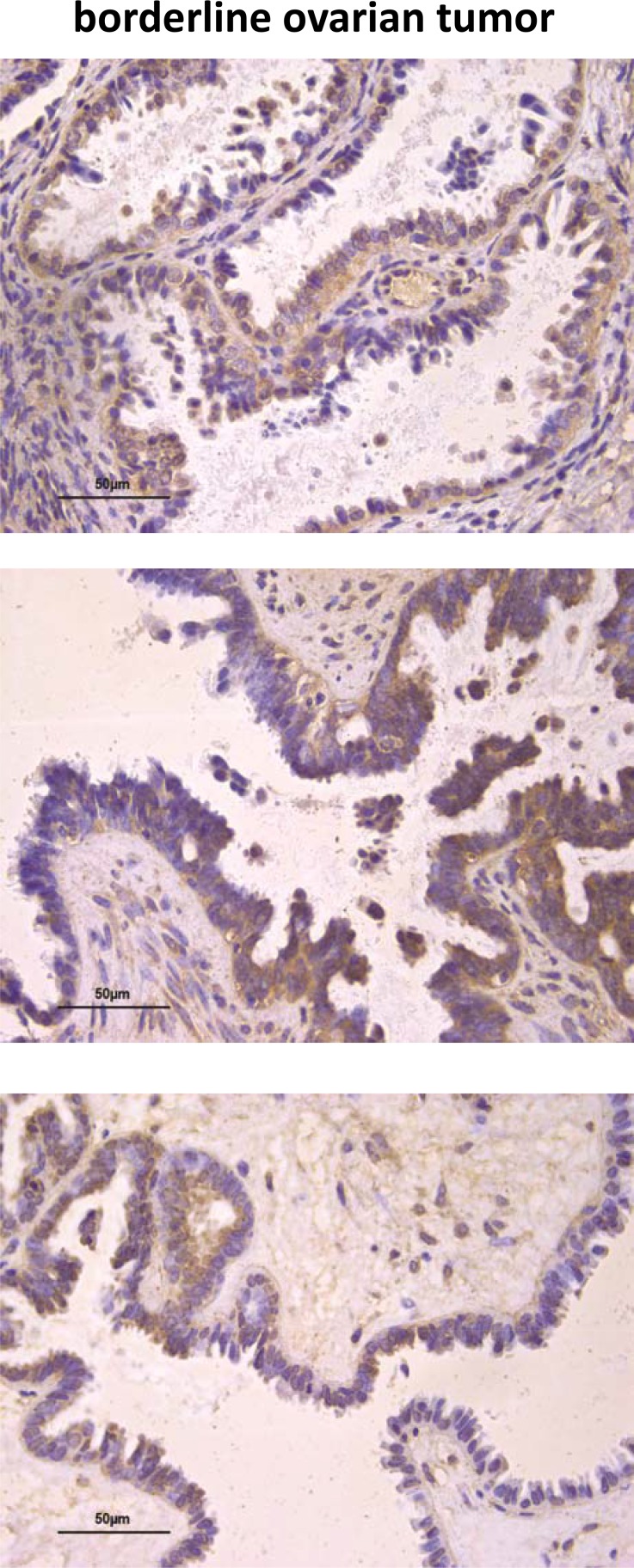
AF1q immunostaining in human ovarian serous BOT Examples of IHC staining for AF1q in BOT tumor cells (upper panel). Example of heterogeneous protein staining in BOT cells (middle panel). Negative IHC staining of AF1q in areas of BOT without evidence of atypical epithelium, which becomes detectable in the areas of transition between normal epithelium and atypical epithelial proliferation (lower panel).

Since the results might suggest an association between high AF1q protein expression and tumor aggressiveness, we extended IHC analyses to 8 serous borderline ovarian tumors (BOT, i.e., tumors with low malignant potential) characterized by intermediate pathological and clinical features between benign and malignant ovarian tumors (such as cytoplasmic and nuclear atypia and the absence of stromal invasion) [40]. The tumors showed AF1q staining but most of them (5/8) displayed a significantly lower protein expression level than that of invasive serous tumors (Table 2 and Figure 8 upper panel, *p* = 0.0276). In addition, opposite to what observed in invasive tumors, in 4/8 BOT the protein staining was heterogeneous, with negative and positive areas (or different reaction intensity) in the same case (Figure 8 middle panel). Interestingly, in normal proliferating epithelium (i.e., OSE, cortical inclusion cysts, and cystic adenoma) as well as in the areas of BOT without evidence of atypical epithelium, the protein was undetectable (Figure 8 lower panel), whereas in the areas of transition between normal epithelium and atypical epithelial proliferation, the protein became detectable (Figure 8 lower panel). All this data together suggested that AF1q level could be related to tumor aggressiveness and might play a role in the ovarian tumorigenic process.

**Table 2:** AF1q expression in BOT versus malignant tumors

Variables	Patients *n* = 39	AF1q		*p* value
		Low (%)	High (%)	
Serous BOT	8	5 (62.5)	3 (37.5)	**0.0276**
Serous Invasive ovarian cancer	31	6 (19.4)	25 (80.6)	

Data in bold indicate *p* < 0.05

### *In silico* validation of the relationship between AF1q and tumor aggressiveness

The relationship between AF1q expression and ovarian tumor aggressiveness was further investigated *in silico* by evaluating its expression level in the Tothill dataset [41] initially focusing on gene expression profiles of invasive ovarian tumors (both serous and endometrioid histotypes). In agreement with IHC analyses, no statistically significant differences were found between AF1q expression and tumor stage (*p* = 0.292, considering each stage separately, Supplementary Figure 4A). On the contrary, we found a significant association between AF1q expression and tumor grade (*p* = 0.031; Supplementary Figure 4B).

We finally investigated whether AF1q expression levels were associated with patients’ outcome, but no significant association was found neither in univariable analysis nor after adjusting or stratifying for the debulking status after surgery (Supplementary Table 3).

**Figure 9:**
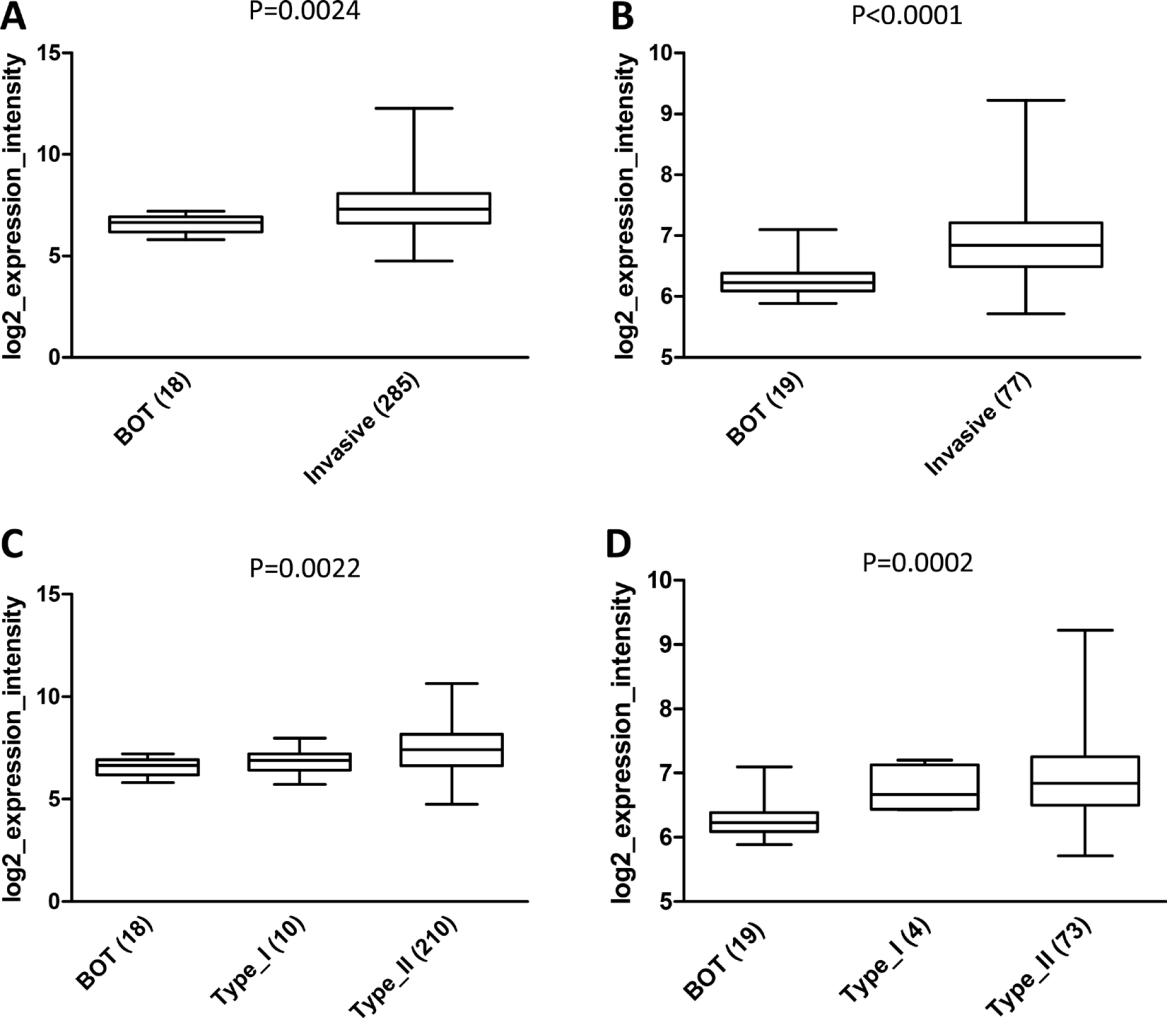
*In silico* analysis of AF1q expression in the Tothill and Berchuck datasets (**A**) and (**B**) Boxplots showing AF1q mRNA expression in BOT compared to invasive tumors in Tothill (*p* = 0.0024 ) and Berchuck (*p* < 0.0001) datasets, respectively. (**C**) and (**D**) Boxplots showing AF1q mRNA expression in BOT and invasive tumors taking into account the pathological dualistic classification of type I and type II in Tothill (*p* = 0.0022) and Berchuck (*p* = 0.0002) datasets, respectively. Numbers in brackets indicate the number of analyzed cases in each group.

Analysis of the Tothill dataset was then extended to BOT. This group showed a statistically significant lower AF1q expression compared to invasive tumors (*p* = 0.0024; Figure 9A). The result was further confirmed in an independent dataset generated by Berchuck et al. [42] which includes only gene expression data from serous ovarian tumors (*p* < 0.0001; Figure 9B). Interestingly, when BOT were compared to invasive tumors classified into type I and II, the differences in AF1q expression among the three groups was still statistically significant (*p* = 0.0022 in Tothill and *p* = 0.0002 in Berchuck datasets; Figure 9C and 9D).

These findings confirmed IHC results, suggesting that up-regulation of AF1q might be involved in the progression from BOT to invasive disease and acquisition of an invasive phenotype; however, variations in AF1q expression within high grade tumors did not correlate with an increased risk of development of metastasis, as suggested by the lack of association with tumor stage and patient outcome.

## DISCUSSION

The present study investigated, for the first time, the oncogenic potential of AF1q in ovarian cancer. We provided evidence that sustained AF1q overexpression increased the aggressive behavior of ovarian cancer cells *in vitro* and that AF1q expression was positively correlated with tumor aggressiveness in clinical ovarian cancer specimens.

The biological activity of AF1q has not yet been clarified; however, based on its first identification as a fusion partner of the mixed-lineage leukaemia protein [1] and considering its involvement in various chromosomal rearrangements in hematopoietic malignancies [2, 4–7, 19], the gene has originally been regarded as an oncogenic factor. Moreover, the notion has been corroborated by other clinical and experimental observations that link the overexpression of AF1q to high tumor aggressiveness and to tumor progression, even in the absence of specific chromosomal aberrations [8, 10–12]. On the other hand, AF1q has been recognized as a protein that mediates the apoptotic activities of certain antitumor agents, such as gamma irradiation and doxorubicin [14, 15]. According to this alternative AF1q function, our group recently reported that its transient overexpression (either drug-induced or obtained by AF1q ectopic expression) caused apoptosis induction in ovarian cancer cells [16]. Thus, AF1q may be ascribed as a protein endowed with a dual behavior, being characterized by both proapoptotic and protumorigenic functions.

In an attempt to clarify this intriguing issue, we investigated whether AF1q, besides its involvement in apoptosis induction, was also endowed with protumorigenic potential in ovarian cancer cells. To this aim, we evaluated the effects of AF1q stable overexpression in the A2780 human ovarian cancer cell line, in order to assess the putative AF1q function in the same cellular context in which we found that its transient transfection induced apoptosis [16].

Our *in vitro* results clearly indicated an association between stable AF1q overexpression and increased tumor aggressiveness and suggested an involvement of the protein in the progression of ovarian cancer. In fact, stable enforced AF1q expression conferred a more aggressive phenotype to A2780 ovarian cancer cells, displaying an elongated shape coupled with modifications in cytoskeleton organization (i.e., distribution pattern of vimentin and actin fibers) and an increase in motility and migratory/invasive abilities. Such features were consistent with distinct molecular alterations that occur during EMT as indicated by the up-regulation of EMT-related transcription factors (Snai1, Snai2 and Zeb1), the reduced expression of epithelial markers (cytokeratins 8 and 18) and the simultaneous up-regulation of mesenchymal markers (vimentin and fibronectin). Through gain and loss of AF1q function experiments in two other cell lines, OVCAR-5 and TOV-21G, we obtained confirmation that the role of the protein in EMT was not restricted to A2780 cells but could be reliably considered a more general mechanism.

The present study was not specifically designed to molecularly dissect the mechanisms underlying AF1q-mediated effects, however, our findings provided some initial insights into its interaction network in ovarian cancer cells. Specifically, we found that AF1q expression level was not affected when cells were forced to undergo MET by transfection of two miR-200 family members (miR-141 and miR-200c) that induced an epithelial phenotype by targeting ZEB1 translation [38], a repressor of E-cadherin [39]. This finding indicated that AF1q was not regulated by ZEB1 but, more likely, acted as an upstream regulator in the EMT process. In addition, molecular pathways affected by AF1q were explored by performing gene expression profiling and GSEA after overexpression of the protein. Not surprisingly, the analysis highlighted a role of AF1q in EMT, a key biological process executed by tumor cells to increase their aggressiveness and acquire invasive features [43]. Through the regulation of cell-cell and cell-matrix adhesions, the phenomenon contributes to the plasticity of ovarian cancer cells, thereby increasing their motility and metastatic potential, and eventually contributing to poor patient outcome [44–46]. In keeping with our findings, a functional role of AF1q in tumor progression has also been reported in human breast cancer [10–12]. In particular, in a recent report, the protein was shown to induce a more aggressive and metastatic behavior of breast cancer cell lines, due, at least in part, to the enhancement of cell motility and migratory/invasive abilities [12], acting as a cofactor for Wnt and STAT signaling pathways. In line with the described relationship between AF1q and Wnt, our data revealed that AF1q overexpression in ovarian tumor cells resulted in positive enrichment of Wnt–β-catenin gene set and involved S100A4 gene, a direct target of this signaling pathway involved in tumor progression and metastasis formation, in ovarian tumor [47]. Intriguingly, the decreased cisplatin sensitivity we observed in AF1q-overexpressing ovarian cancer cells is in line with the chemoresistance phenotype conferred by the protein to breast cancer cells [12] and with the knowledge that EMT plays a critical role in cancer drug resistance [20]. It is also interesting to note that a specific link between expression levels of S100A4 and cisplatin sensitivity has been reported [22].

Gene expression analysis also showed a positive enrichment of other gene sets, including Ras pathway, response to hypoxia and to estrogen, previously described to crosstalk with MAPK transduction cascade [35–37]. This finding prompted us to analyze MAPK activation status revealing an increase in phosphorylation of ERK and p38 proteins that could represent a potential additional mechanism by which AF1q induces EMT in ovarian tumor cells. However, whether AF1q directly or indirectly affects the MAPK pathway requires further research.

Our *in vitro* evidence is corroborated by the results obtained in ovarian cancer specimens, in which IHC and *in silico* analyses for the expression of AF1q (protein and mRNA, respectively) demonstrated a link between high AF1q expression and tumor malignancy. In fact, as assessed by IHC analysis, the AF1q staining was undetectable in normal ovarian epithelium, becoming positive when atypical proliferation was present. Moreover, a differential AF1q expression (both at protein and mRNA levels) between tumors with low malignant potential (i.e., BOT) and invasive ovarian cancers was observed, being significantly higher in the latter than in the former. Interestingly, this particular result was especially in line with *in vitro* findings associating the overexpression of AF1q with the activation of the EMT process and the acquisition of a more migratory/invasive behavior. In fact, the main difference between BOT and malignant ovarian tumors is represented by the different invasive ability, since BOT are defined as non-invasive tumors. It is noteworthy that the relationship between AF1q and aggressive tumor behavior persisted taking into account the pathological classification of type I and II ovarian cancers. All these findings, coupled with the association observed between AF1q level and tumor grade, supported a potential role of AF1q in ovarian tumor progression. According to our results, IHC analysis in breast cancer specimens revealed that AF1q was not present in normal epithelial cells and that its expression became detectable in cancerous cells, being more intense in metastatic sites [12]. In addition, in the same study, high AF1q expression was found to be positively associated with poor overall, disease-free, and metastasis-free survival of breast cancer patients. However, we did not find a significant association with tumor stage or patients’ outcome, suggesting that AF1q role in ovarian cancer is primarily related with the acquisition of an invasive phenotype in the primary tumor rather than with a direct promotion of development of distant metastasis. Accordingly, an *in silico* analysis of the gene expression dataset GSE30587 [48] which included 18 samples from 9 matched pairs of primary ovarian tumors and omentum metastases did not reveal statistically significant differences in AF1q expression between the two entities (data not shown).

The present study did not thoroughly investigate the molecular events underlying AF1q protumorigenic activity in ovarian cancer. Nevertheless, we can speculate that, by acting as a co-factor [12, 13], AF1q could have a pleiotropic influence on the transcription of multiple genes through the interaction with different transcription factors, thus allowing the switch between proapoptotic and protumorigenic programs. In alternative to this hypothesis, as previously described for certain well-characterized oncogenes [49], it is conceivable that the induction of apoptosis upon oncogenic stimuli could be a response evoked by cells as self-defense mechanism to counteract aberrant cell growth. Such defense programs have been shown to be activated as a result of the cellular stress caused by aberrant oncogene activation as a protective barrier to prevent the emergence of potentially harmful cells [49]. Thus, escaping fail-safe mechanisms could represent a determinant step in driving cell transformation, tumor initiation and progression. In line with this hypothesis, we cannot exclude that also the stable AF1q transfection did not initially cause an induction of apoptosis, possibly covered by the cell death provoked by the antibiotic selection.

In conclusion, the present study demonstrated, for the first time, that AF1q is endowed with protumorigenic activity in ovarian cancer. Together with our previous observations related to the proapoptotic function of the protein, such findings highlighted a dual behavior of AF1q in this malignancy. Taken together our results demonstrated a role for AF1q in cellular migration and invasion and in the acquisition of tumor invasive and aggressive features, thus pointing out an involvement of the protein in ovarian tumor progression. Understanding the molecular bases underlying the role of AF1q in ovarian tumor progression absolutely requires and deserves further investigation, in order to determine whether targeting the protein or its regulatory axis could be a useful therapeutic strategy for the malignancy.

## MATERIALS AND METHODS

### Cell culture and transfection

The ovarian tumour cell lines A2780 (obtained from Dr. Ozols, Bethesda, MD), OVCAR-5 (obtained by Dr. Camalier, NCI-NIH) and TOV-21G (purchased from ATCC, Manassas, VA), were cultured in RPMI 1640 (Lonza, Basel, Switzerland) containing 10% foetal calf serum at 37°C under 5% CO_2_. To establish stable transfectants, A2780 cells, OVCAR-5, and TOV-21G were seeded (5 × 10^5^), in 60 mm dishes and 24 h later, a mixture of Lipofectamine 2000 reagent (Invitrogen, Carlsbad, CA) and 8 μg of expression or silencing plasmid was added and incubated for 6 h. Cells were subsequently cultured in medium supplemented with 10% serum for an additional 48 h before adding G418 (Gibco Brl, Paisley, UK) at a concentration of 400 μg/ml for A2780 and OVCAR-5 cells or Puromycin (Sigma Aldrich, St Louis, MO) at a concentration of 2.5 μg/ml for TOV-21G, used for the selection of transfectant clones. miR-200c and miR-141 mimics were transiently transfected in A2780 and TOV-21G cells. Cells were seeded and transfected according to the above mentioned transfection protocol using 20 μM miR-200c mimic (Thermo Fisher) and 20 μM miR-141 mimic (Thermo Fisher) and analyzed after 72 hours.

### AF1q expression and silencing plasmids

Two different constructs were used to overexpress AF1q: a GFP-tagged AF1q expression vector pEGFP-N1 constructed as described previously [16] and AF1q1 full-length cDNA cloned into the expression vector pcDNA3.1 (Clontech, Palo Alto, CA). For the latter, AF1q open reading frame was obtained by Polymerase Chain Reaction (PCR) using forward primer 5′-GAATTCCCACCATGAGGGACCCTGTGA-3′ (containing ECORI restriction site and Kozak sequence CCACC) and reverse primer 5′- CTCGAGTTAGAGCAAGTCCAGTTCGAAG-3′ (containing Xho restriction site). Plasmids expressing an AF1q shRNA and a scrambled non-silencing siRNA were purchased from Origene (Rockville, MD, #TF319110).

### Growth inhibition assay

Cells were seeded at a density of 7000 cells per well in 96 cluster tissue culture plates, treated the next day with increasing concentrations of cisplatin (from 0.05 μM to 0.8 μM), and incubated for 72 hours. Cell growth inhibition was estimated by using the sulforhodamine B assay. Three analyses were performed, and four replicate wells were used for each analysis. IC_50_ values were calculated by interpolation of the sigmoidal dose response curves.

### Immunoblot analysis

Proteins were extracted by lysing cells in sodium dodecyl sulfate (SDS) sample buffer (62.5 mM Tris–HCl [pH 6.8], 2% SDS) containing 1 mM phenylmethylsulfonyl fluoride, 10 μg/mL pepstatin, 12.5 μg/mL leupeptin, 2 μg/mL aprotinin, 1 mM sodium orthovanadate, and 1 mM sodium molybdate. Cell extracts were processed for western immunoblotting as described previously [50]. The following antibodies used for immunoblotting were purchased from the indicated suppliers: mouse monoclonal antibody against AF1q from Abnova (Taipei City, Taiwan), mouse monoclonal antibody against GAPDH from Sigma Aldrich (St Louis, MO), mouse monoclonal antibody against cytokeratin 8/18 from Santa Cruz Biotechnology (Santa Cruz, CA), rabbit polyclonal antibody against Fibronectin from Sigma Aldrich, mouse monoclonal antibody against Vimentin from Abcam (Cambridge, UK), rabbit polyclonal antibody against p38 from Santa Cruz Biotechnology, rabbit polyclonal antibody against Thr180/Tyr182 p38 phosphorylations from Cell Signaling (Danvers, MA), rabbit polyclonal antibody against Erk1/2 from Sigma Aldrich, rabbit polyclonal antibody against Thr202/Tyr204 Erk1/2 phosphorylations from Cell Signaling.

### Immunofluorescence analysis

Cells, grown on glass coverslips slides in 24 mm Petri dishes, were fixed in 4% paraformaldehyde at room temperature for 10 minutes, permeabilized with 0.2% Triton X-100 at room temperature for 5 minutes, washed with PBS and then stained with fluorescent phalloidin (Sigma Aldrich) or Vimentin (Abcam) followed by the secondary antibody anti-mouse Alexa 488 (Molecular Probes, Eugene, OR). Slides were fitted with Fluoroshield mounting medium with DAPI (Abcam) and analyzed with a fluorescence microscope [images were recorded with a Spot Insight digital camera (Delta Sistemi, Rome, Italy) equipped with a system of image analysis (IAS 2000; Delta Sistemi)].

### Wound-healing assay

Cells (1.5 × 10^6^) were plated in six-well plates, grown until they reached approximately 90% confluence, and starved for 24 h in serum free medium. The culture medium was then removed and the cell monolayer was wounded with a sterile pipette tip and washed twice with PBS. Subsequently, fresh culture medium supplemented with 1% of serum was added, and the cells were allowed to close the wound for 48 h. Photographs of the same wound position were taken over time after scratching and the migration ability of the cells was determined by measuring the width of the wound. The experiment was performed in triplicate.

### Cell migration and invasion assays

Both migration and invasion assays were performed in 24-well dishes using Transwell inserts with 8-μm sized pores (Corning Costar, NY). In the case of invasion assay, filters were pre-coated with 60 μL Matrigel (BD Biosciences, Bedford, MA) for 30 minutes. After 24 h of starvation, cells were trypsinized and re-suspended in serum-free medium at a density of 0.4 × 10^6^ cells/ml, and 300 μl of this cell suspension was seeded to the upper side of the filters and 1 ml of serum-supplemented medium was placed in the lower chamber (for invasion assay the density used was 0.8 × 10^6^ cells/ml). Cells were permitted to migrate for 48 h and, after incubation, stationary cells were removed from the upper surface of the membranes using a cotton swab. The cells on the lower membrane surface were fixed with ethanol and stained with sulforhodamine B. The number of stained cells in five randomly chosen fields was counted under a light microscope. The experiment was repeated three times.

### RNA extraction and real-time PCR

For gene expression Real-Time PCR analysis, single-stranded cDNA was synthesized from 2 μg of total cellular RNA extracted using Trizol reagent (Thermo Fisher) according to the manufacturer's instructions. The synthesis was performed by using the High Capacity RNA-to-cDNA Kit (Thermo Fisher) according to the manufacturer's instructions. EMT-related transcription factors, such as Snai1, Snai2, Zeb1, Fn (fibronectin), Cdh1 (E-cadherin) and Cdh2 (N-cadherin) levels were analyzed by Real-Time PCR using specific TaqMan assays (Applied Biosystems; assay IDs: Hs00195591_m1, Hs00161904_m1, Hs00232783_m1, Hs01549976_m1, hs01023894_m1 and Hs00983056_m1, respectively) and the ABI PRISM 7900 HT Sequence Detection System (Applied Biosystems). All the data were analyzed by SDS 2.4 software (Applied Biosystems) and reported as relative quantity (RQ) with respect to Mock1 expression considered as calibrator sample using comparative Ct method (ΔΔCt), in which GAPDH (assay ID: Hs02758991_g) expression was used to normalize raw Ct data (obtaining ΔCt).

For Real-Time PCR analysis of miR-141 and miR-200c, complementary DNA was synthesized from 5 ng of total RNA, after extraction with the commercial column-based system Qiagen miRNeasy^®^ Mini Kit (Qiagen, Valencia, CA, USA), using the TaqMan microRNA Reverse Transcription Reagents (Thermo Fisher) according to the manufacturer's instruction and using assay 000463 and 002300 (Thermo Fisher). Data analyses was performed using ΔΔCt method in which U6 (assay ID: 001973) expression was used as internal calibrator.

### Microarray experiments and data processing

Total RNA, after a clean-up treatment with RNAeasy kit (Qiagen, Valencia, CA) and with RNase-free DNase to remove contaminating genomic DNA, was assessed for integrity and purity by Bioanalyzer (Agilent, Santa Clara,CA).

RNA samples were processed for microarray hybridization by the Functional Genomics core facility at the Fondazione IRCCS Istituto Nazionale dei Tumori of Milan. Briefly, 500 ng of total RNA was reverse transcribed, labeled with biotin and amplified overnight using the Illumina RNA TotalPrep Amplification kit (Ambion) according to manufacturer's protocol. One ug of the biotinylated cRNA sample was mixed with the Hyb E1 hybridizatioin buffer containing 37.5% (w/w) formamide and then hybridized to Illumina HumanHT-12v4 chips (47,324 probes) (Illumina, Inc., San Diego, CA) at 58°C overnight. Array chips were washed with manufacturer's E1BC solution, stained with 1 ug/ml Cy3-streptavidine (Amersham Biosciences) and eventually scanned with Illumina BeadArray Reader. We collected primary data using the supplied scanner software and the following analyses were performed using the BeadStudio Version 3 software package. Raw data was normalized using the RSN normalization as implemented in the *lumi* R/Bioconductor package. Probes with detection *p value* > 0.01 in all samples were discarded before downstream analyses.

Enrichment analysis in mRNA expression data after AF1q stable transfection in A2780 cells was performed using GSEA [51]. The Hallmark collection containing 50 gene sets, representing well-defined biological pathways and states, was tested for enrichment. Gene expression was ranked according to fold change. Gene sets with a FDR < 0.25 were considered significantly enriched.

### Patient specimens

We analyzed 47 specimens from previously untreated patients with invasive ovarian cancer, selected from the Gynecologic Oncology Unit of the Fondazione IRCCS Istituto Nazionale dei Tumori of Milan (from 2004 to 2009) and from the “Sf. Spiridon” Clinical Emergency County Hospital and “Cuza-Vodă” Obstetrics and Gynecology University Hospital of Iassy (from 2009 to 2011) (Table 1). We also included in our analysis 8 specimens from patients with stage I serous BOT selected from the case series of the former Institute. The study was approved by the respective local ethics committees, and patients signed an informed consent to donate the leftover biological material for research studies. Patient records were reviewed: histologic subtypes were evaluated according to the dualistic model (type I and type II), tumor stages were assessed according to the International Federation of Gynecology and Obstetrics (FIGO) standards and tumor grade by the WHO classification.

### Immunohistochemical analysis

Paraffin-embedded formalin-fixed ovarian cancer tissues were sectioned (4 mm thick). Unstained sections were de-waxed and rehydrated. For epitope unmasking, a sodium citrate 10 mM (pH 6.0) procedure based on autoclave treatment for 15 minutes was applied. Blocking of the endogenous peroxidase for 10 minutes was performed by using H_2_O_2_ and 3% methanol. Incubation with the primary antibody anti-MLTT11/AF1q (Epitomics, Burlingame, CA) was done with a working dilution 1:25 overnight at 4°C. The secondary antibody used was a Biotinylated Secondary Antibody (Dako REAL^™^ Link; Dako, Glostrup, Denmark) for 15 minute at room temperature and addition of the high-sensitivity streptavidin-HRP conjugate (Dako REAL^™^ Streptavidin Peroxidase) was performed for 15 minutes. The sections were treated with 3.3′-diaminobenzidine tetrahydrochloride chromogen (Dako REAL™ DAB) for 5 minutes. Nuclei were counterstained with hematoxylin. Lymphoid tissue of thymus was used as an external positive control. The negative control sample (primary antibody omitted) did not show any signal. Immunohistochemical reaction for AF1q was assessed using a semi quantitative score, based on the percentage of positive cells and staining intensity of the reaction. The percentage of positive cells of each sample was scored based on cytoplasmic staining (< 5% or > 5%). Only the samples with staining >5% were considered as tumor expressions. The intensity of cytoplasmic staining of samples was graded into three groups: weak (+), moderate (++) and strong (+++). Based on positive cells and their staining intensity the samples were divided into two groups: low (i.e., negative (0–5%) or > 5% positive cells and weak intensity) and high (i.e., >5% positive cells and moderate or strong intensity).

### *In silico* analysis

The *in silico* analyses were performed on Tothill [23] and Berchuck [24] datasets, which are the two largest publicly available ovarian cancer datasets including both invasive tumors (representative of the different stages and grades) and BOT. Raw data from the Tothill dataset was downloaded from the NCBI Gene Expression Omnibus repository (GSE9891), and the Berchuck dataset was downloaded from the Duke Institute website (www.duke.edu). The former dataset, which was obtained using the Affymetrix platform, was RMA normalized using the proprietary Expression Console software. Upon quality control, for each dataset the probe corresponding to AF1q (i.e., the 211071_s_at) in the current annotation version (na34, www.affymetrix.com) was extracted and the analyses were performed through GraphPad Prism version 5.

### Statistical analysis

Experiments were carried out at least in triplicate. Differences between mean values were assessed by two-tailed Student's *t*-test and *Fisher's exact* test and one-way ANOVA. *p* values < 0.05 were considered as statistically significant. Univariable and multivariable Cox regression analysis (as implemented in the survival R package) was used to correlate AF1q and/or clinico-pathological variables with survival.

## SUPPLEMENTARY MATERIALS FIGURES AND TABLES


