# Supplementary material for: Involvement of AF1q/MLLT11 in the progression of ovarian cancer
Source: Oncotarget. 2017 Feb 21;8(14):23246–64. doi: 10.18632/oncotarget.15574 (PMC5410301; doi:10.18632/oncotarget.15574)
Supplement: Supplementary file 1 [file oncotarget-08-23246-s001.pdf]

Involvement of AF1q/MLLT11 in the progression of ovarian cancer

Supplementary Materials

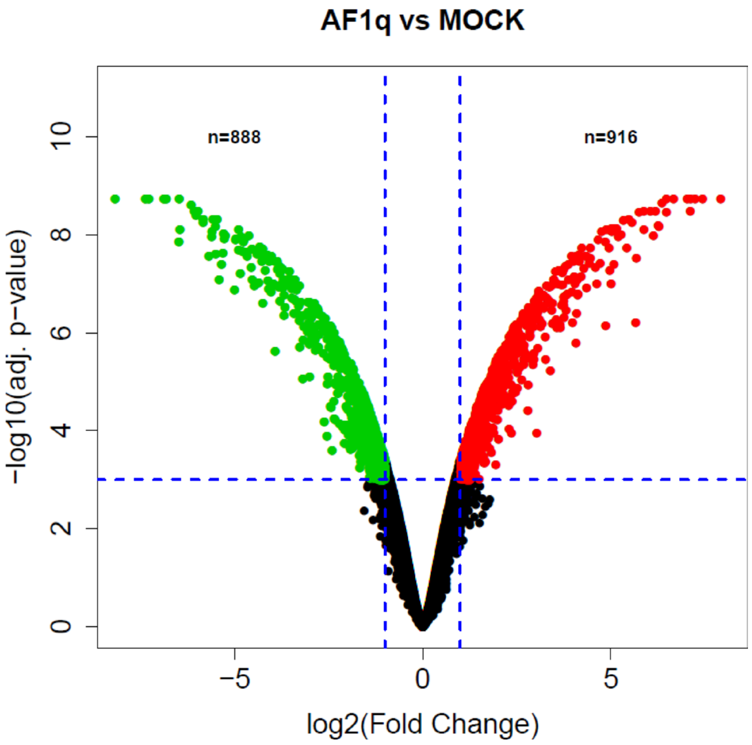

Supplementary Figure 1: Volcano plot summarizing differential analysis resultsin A2780 cells overexpressing AF1q compared to mock cells. For each gene, log2 fold change is plotted on the x-axis and the negative log10 *t*-test *p*-value on the y-axis. Statistical significance threshold (adj $p < 0.001$) is indicated by the horizontal line.

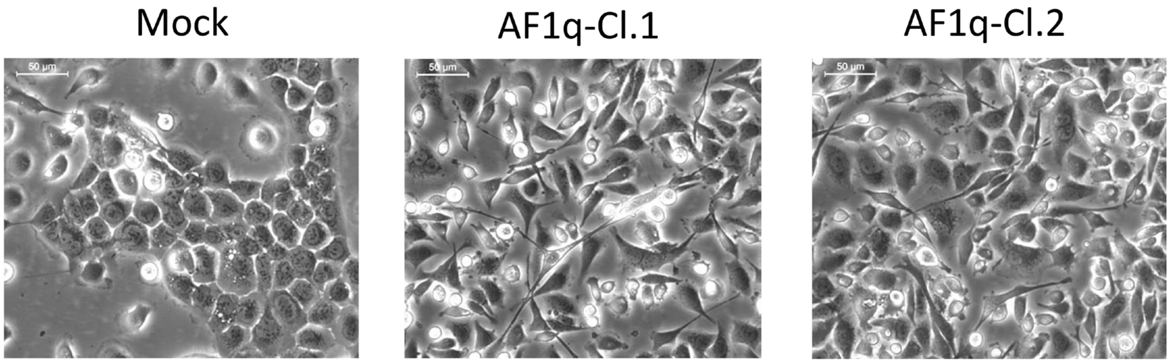

Supplementary Figure 2: AF1q overexpression induces a spindle-shape phenotype in OVCAR-5 ovarian cancer cells. Morphological appearances of mock and AF1q overexpressing OVCAR-5 clones (Cl.1 and Cl.2).

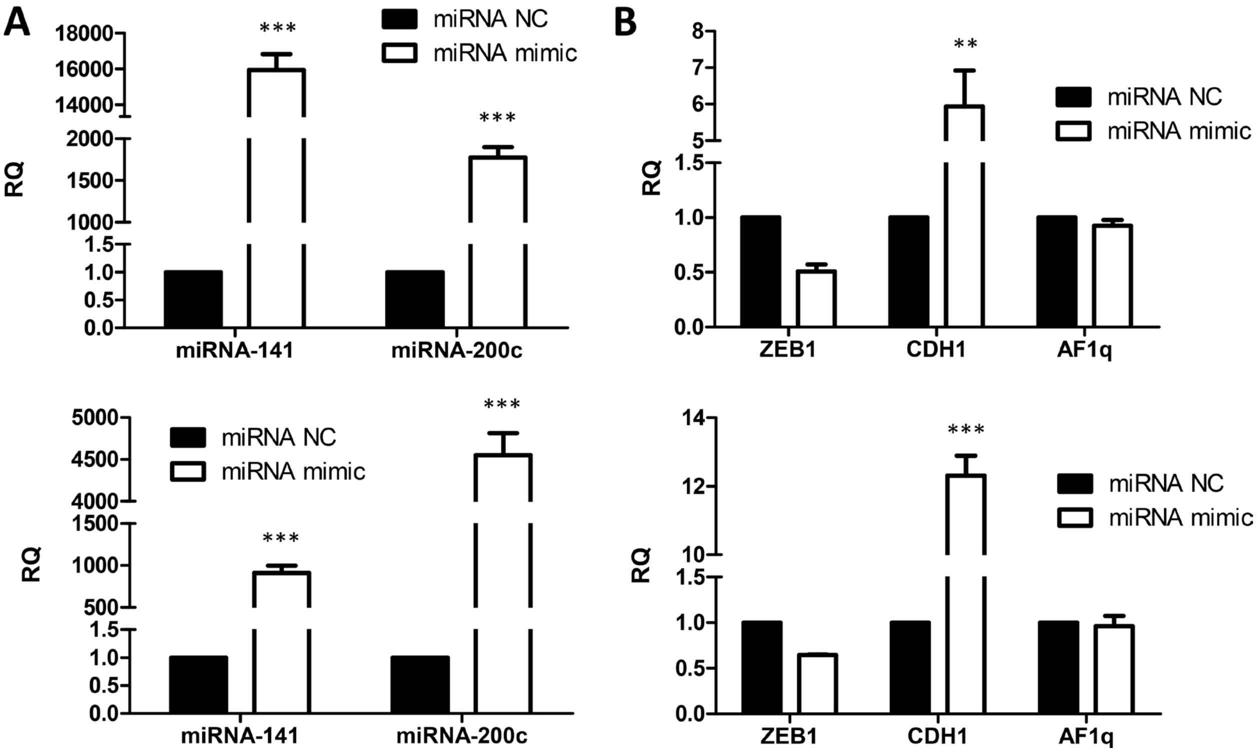

Supplementary Figure 3: Induction of MET by miR-200 family transfection does not affect AF1q expression. (**A**) Real-Time PCR analyses showing miR-141 and miR-200c expression levels 72 h after transfection in TOV-21G (upper panel) and A2780 (lower panel) cell lines compared to miRNA non coding (NC) transfected cells. (**B**) Real-Time PCR analyses of Zeb1, Cdh1 and AF1q in TOV-21G (upper panel) and A2780 (lower panel) miRNA transfected cell lines compared to miRNA non coding (NC) transfected cells. Asterisk indicate *p*-value: ** from 0.001 to 0.01 and ***< 0.001

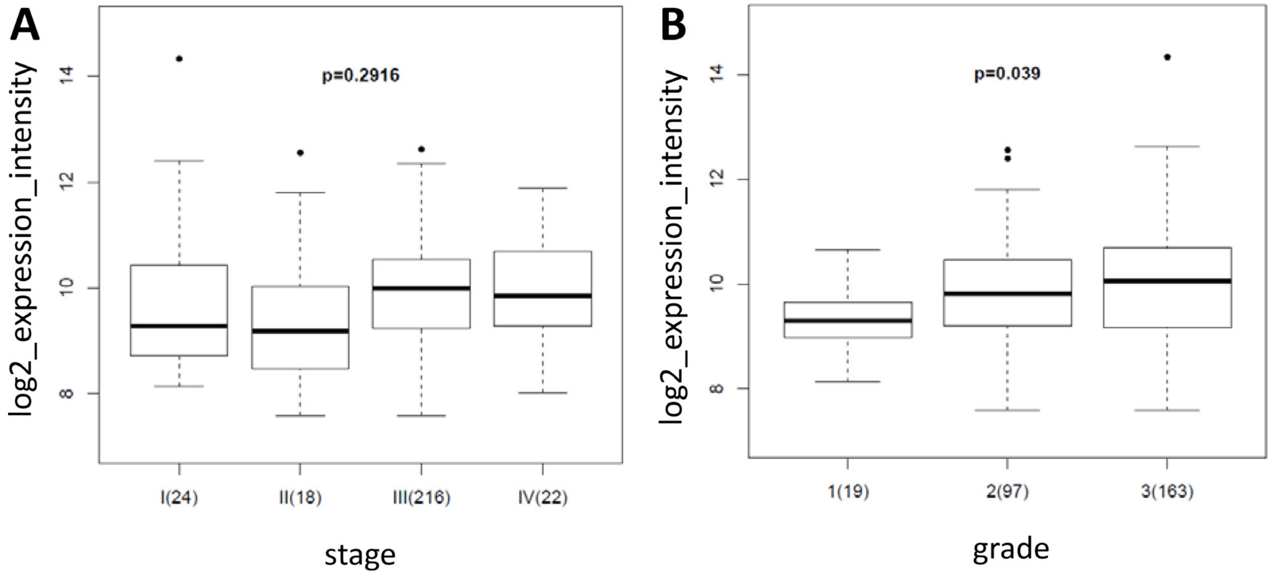

Supplementary Figure 4: Correlation of AF1q expression with tumor stage and grade in the Tothill dataset. Boxplots showing AF1q mRNA expression according to (**A**) tumor stage (p = 0.292) and (**B**) grade (p = 0.031). Numbers in brackets indicate the number of analyzed cases in each group.

Supplementary Table 1: Top 25 up and down-regulated genes in A2780 cells overexpressing AF1q (Cl.9) compared to mock cells

Up-regulated genes	Fold-change	Adjusted *p*-value	Down-regulated genes	Fold-change	Adjusted *p*-value
AKR1C3	241,9	1,86E-09	MDK	0,003	1,86E-09
S100A4	174,0	1,86E-09	SPP1	0,006	1,86E-09
TMSL3	151,2	1,86E-09	CBR3	0,006	1,86E-09
AKR1C2	139,5	1,86E-09	CYBA	0,009	1,86E-09
DKK1	138,8	3,24E-09	LOC731042	0,009	1,86E-09
PI15	131,1	1,86E-09	PCDH19	0,011	1,36E-08
AKR1C4	101,6	1,86E-09	LIN28	0,011	1,86E-09
TMEM166	89,18	1,86E-09	POU4F1	0,011	7,91E-09
NFIB	82,22	2,23E-09	SPAG6	0,014	2,52E-09
CH25H	77,57	6,70E-09	DIRAS3	0,015	3,24E-09
MGP	76,63	6,30E-09	DLK1	0,015	3,24E-09
MMP3	73,05	3,24E-09	GPM6B	0,015	3,94E-09
ACTG2	70,24	1,04E-08	HS.19193	0,016	3,24E-09
MARCKS	66,36	3,24E-09	TFAP2A	0,018	4,76E-09
PRPH	58,88	7,97E-09	TFPI	0,018	5,53E-09
LOC100129681	58,87	3,24E-09	RFTN2	0,018	4,76E-09
TNFSF10	53,92	3,38E-09	RSPO3	0,020	2,75E-08
TSPAN8	51,32	2,96E-08	C3ORF70	0,020	1,08E-08
CRYAB	50,51	6,05E-07	ZIC2	0,021	8,53E-09
ISL1	47,14	5,68E-09	FAM5C	0,021	4,76E-09
SOX18	44,64	4,76E-09	RBM47	0,022	6,70E-09
SLC44A1	43,08	1,88E-08	CHRNA9	0,022	2,51E-08
BST2	40,26	5,12E-09	C19ORF30	0,023	4,90E-09
CD36	38,61	9,93E-09	VCAN	0,024	8,09E-08
MMP10	36,69	1,11E-08	SEMA6A	0,025	4,08E-08

Supplementary Table 2: Summary of the enriched gene sets with FDR < 0.25 obtained through the GSEA analysis

	Positively enriched Gene sets	Size	NES	FDR *q*-val
1	HALLMARK_MYOGENESIS	118	2.14	0.000
2	HALLMARK_ EPITHELIAL_MESENCHYMAL_TRANSITION	139	1.94	0.001
3	HALLMARK_KRAS SIGNALING_UP	109	1.83	0.002
4	HALLMARK_WNT_BETA_CATENIN_SIGNALING	33	1.80	0.002
5	HALLMARK_COAGULATION	78	1.79	0.002
6	HALLMARK_P53_PATHWAY	169	1.73	0.006
7	HALLMARK_ESTROGEN_RESPONSE_EARLY	133	1.72	0.006
8	HALLMARK_ESTROGEN_RESPONSE_LATE	138	1.61	0.021
9	HALLMARK_HYPOXIA	158	1.59	0.025
	**Negatively enriched Gene sets**	**Size**	**NES**	**FDR *q*-val**
1	HALLMARK_APICAL_SURFACE	22	−1.66	0.046

Size indicates the number of genes included in the gene set;
NES indicates the normalized enrichment score (enrichment score for the gene set after normalization across analyzed gene sets);
FDR *q*-val indicates the false discovery rate.

Supplementary Table 3: Univariable and multivariable cox regression analysis in malignant serous samples from the Tothill dataset

	RFS		OS	
**Variable**	**HR**	***P***	**HR**	***P***
AF1q (Univariable)	0.96	0.635	0.96	0.635
Debulking (Univariable)	1.79	0.007	1.47	0.074
Multivariable analysis AF1q	0.92	0.414	0.95	0.564
Debulking (> 1 vs ≤ 1)	1.81	0.006	1.48	0.069
